# Clinical evaluation of negative mNGS reports in sterile body fluids and tissues

**DOI:** 10.1128/spectrum.02013-24

**Published:** 2025-03-25

**Authors:** Wang Lisha, Qian Jiao, Chen Mengyuan, Qin Jiajia, Tang Tianbin, Wang Yilan, Hu Linjie, Yu Sufei

**Affiliations:** 1 Department of Laboratory Medicine, Taizhou Hospital of Zhejiang Province affiliated to Wenzhou Medical University 56709 , Linhai, China; 2 Key Laboratory of System Medicine and Precision Diagnosis and Treatment of Taizhou, Taizhou, China; 3 Health and New Drug Clinical Research, Taizhou Institute of Medicine, Taizhou, China; 4 Department of Laboratory Medicine, Enze Hospital, Taizhou Enze Medical Center (Group) 659328 , Taizhou, China; National Chung Hsing University, Taichung, Taiwan, China

**Keywords:** mNGS, sterile body fluids and tissues, true negative, clinical relevance, clinical impact

## Abstract

Regarding metagenomics next-generation sequencing (mNGS) negative literature, there have been much discussions about methodology; thus, we collected clinical and laboratory information for retrospective evidence-based research. We retrospectively analyzed 150 negative mNGS results of patients suspected of having aseptic body fluid infections and divided them into a plasma group, cerebrospinal fluid (CSF) group, and others group according to the sample types. Based on the final laboratory and clinical diagnoses, the diagnostic accuracy of excluding infections in the plasma, CSF, and others groups of negative mNGS results were 72.0%, 40.4%, and 30.2%, respectively. The false-negative rates of the CSF and others group were relatively high. The positive impact rates of clinical application in the plasma, CSF, and others groups were 68.0%, 40.4%, and 25.6%, respectively. Three factors, including patient department distribution, admission symptoms, and doctors' judgment of patient infection were used to analyze the reasons for uncertain negative or false-negative results in mNGS. The clinical information analysis of false-negative patients’ aims were to reduce the false-negative rate and improve the diagnostic accuracy of mNGS. On the selection of sampling timing in mNGS, within half a month after a patient develops suspected symptoms of infection, the earlier the mNGS test, the higher the true-negative rate.

**IMPORTANCE:**

There has been little research carried out on the diagnostic value of negative metagenomics next-generation sequencing (mNGS) results in clinical practice, especially for sterile body fluids. In the present study, plasma negative mNGS results showed the highest diagnostic accuracy for excluding infection. However, the cerebrospinal fluid and other mNGS false-negative rates were 59.6% and 69.8%, respectively. Our findings emphasized the role of negative mNGS results in practical clinical applications and clarified that patients, mNGS sampling time, and doctor’s decision making were the key factors for the diagnosis of clinical infections. More attention should be paid to the diagnostic role of mNGS true negatives, the analysis of clinical patterns of false negatives, and improving the diagnostic accuracy of mNGS.

## INTRODUCTION

Infectious diseases refer to diseases that are responsible for human infections and clinical symptoms caused by pathogenic microorganisms. The death toll of infectious diseases accounts for 28% of total deaths worldwide (1). Pathogen detection technology based on cultivation as the gold standard can no longer meet clinical diagnostic requirements, so molecular diagnostic technology has been rapidly developed. Rapid and accurate etiological diagnosis can help clinicians optimize the use of antibiotics in a timely manner, thereby reducing the length of the hospital stay for patients, improving the cure rate and prognosis (2). Moreover, when the patients' clinical symptoms fall between infection and other diseases, efficient and accurate detection techniques can assist doctors in making a clear diagnosis (3, 4). Metagenomics next-generation sequencing (mNGS) is increasingly used in clinical laboratories to diagnose complex diseases and has the advantages of high throughput and detection of non-culturable and trace microorganisms (5, 6).

Currently, mNGS technology depends on professional bioinformatics teams and can meet personalized bioinformatics analysis requirements (7, 8). In a study published by Miller et al. in 2019, mNGS was used to detect 95 cerebrospinal fluid (CSF) infections and showed 73% sensitivity. A total of 21 cases were classified as mNGS false negatives. The reasons for false negatives can be summarized as follows: first, some pathogens are high-background samples that are prone to missed detection and low viral load in viral infection; second, some special pathogenic bacteria, such as *Sporothrix suis*, are not included in the known database (9). A study reported the diagnosis and treatment process of three rare patients with invasive mold fungal diseases of the central nervous system. One patient had multiple negative CSF mNGS tests, and finally, *Aspergillus fumigatus* was detected in the drainage fluid of a brain abscess. Subsequently, *Aspergillus fumigatus* growth was also detected in culture (10). This can also indicate that the reason for the false negative is the selection of the specimen type, and it can also signify that the earlier the diagnosis of infectious disease is made, the earlier it can be cured. Another study analyzed the risk factors for culture negativity in bone and joint infections using mNGS and pointed out that invasive osteoarticular infection (IOI), multi-infections, rare pathogen infections, and prior antibiotic use are risk factors for negative microbial cultures. Therefore, the risk factors for mNGS false negatives are also worth exploring (11). In 2023, we published a clinical application analysis on plasma mNGS tests that involved the detection performance and diagnostic performance of mNGS, emphasizing the positive impact of clinically consistent mNGS positive detection on clinical diagnosis and treatment (12). But the paper lacked an in-depth analysis of negative mNGS results.

Thus, the present study focused on analyzing the correlation between negative mNGS results and the condition in patients with suspected aseptic fluid infections, as well as the actual clinical impact. It deeply explored several influencing factors of negative mNGS results, mainly including the patient’s admission symptoms and department distribution, the timing of mNGS testing, and the doctor’s judgment of the patient’s condition.

## MATERIALS AND METHODS

### Study population and ethics

From 1 July 2021 to 12 May 2023, Taizhou Enze Medical Center (Group) conducted a total of 1,499 mNGS tests. There were 791 mNGS tests for different types of sterile body fluids and tissues, of which 153 had negative mNGS results. Three negative mNGS results of patients with incomplete information were excluded from the study, and the remaining 150 negative mNGS results of patients were included. Data of each case were unrepetitively collected through the electronic medical record system. This study was approved by the Ethics Committee of Taizhou Hospital in Zhejiang Province, China (No. K20230862).

### Definitions

#### Infection

Similar to related research (13–15), the treatment team (composed of at least three attending physicians experienced in infectious diseases) made the comprehensive judgment of a clinical infection diagnosis based on the patient’s current infection indicators, pathogenic testing (including cultures, antigen tests, antibody tests, histopathology tests, PCR, mNGS tests), imaging data, treatment status, and clinical symptoms.

#### True negative, false negative, and uncertain negatives

The treatment team decided if the patient had an infection in sterile body fluid or tissue sites based on infection diagnostic criteria. In cases where the diagnostic sampling site was not infected, an mNGS negative result was defined as true negative. Conversely, in cases of infection at the diagnostic sampling site, an mNGS negative result was defined as false negative. However, if the treatment team suspected the presence of a bloodstream infection without a clear diagnosis, then, we defined plasma negative mNGS results as uncertain negative.

#### Negative predictive value

Clinical diagnosis served as a reference for evaluating the negative predictive value of mNGS tests. The negative predictive value = the number of true negatives/(number of true negatives + the number of false negatives [or uncertain negatives]).

#### Clinical impact of negative mNGS results

In the present study, we retrospectively analyzed timely feedback from treatment teams on negative mNGS results using the electronic medical record system and divided the clinical impact of negative mNGS results into a positive impact and no impact. We defined a positive impact of negative mNGS results as the treatment team ruling out an infection at the mNGS sampling site. The definition of no impact was that the treatment team was still considering infection, or the patient was discharged or died before the mNGS results were reported. The specific categories of each type of clinical impact and its effect on patient outcomes are shown in Table 2.

#### The sampling time of mNGS

Depending on the sampling time of mNGS after the appearance of suspected infection symptoms, we divided patients into groups based on the time from the appearance of suspected infection symptoms to the sampling time of mNGS, with every 5 days divided into a group, including ≤5 days (0–5), >5 or ≤10 days (5–10), >10 or ≤15 days (10–15), >15 or ≤20 days (15–20), >20 days (>20), for a total of five groups.

### Sample testing

#### mNGS testing

##### mNGS sampling and pre-processing

The sterile body fluids and tissues in this study include plasma, CSF, pleural effusion, ascites, pericardial effusion, joint fluid, tissue fluid, and tissues. Blood collection personnel took blood samples of 3 mL from adults and 1 mL from children, placed them in K2 ethylene diamine tetraacetic acid anticoagulant tubes, which were then centrifuged and frozen for subsequent analysis. Specialist doctors standardized the collection of the second tube of CSF (1 mL), pericardial effusion (1 mL), tissue fluid (1 mL), pleural effusion (3 mL), ascites (3 mL), joint fluid (0.5 mL), and green bean-sized tissues were placed in sterile containers. Then, after physically grinding the tissue samples, we took the approximate size of rice grains and placed them in an Eppendorf tube. Glass beads and nuclease-free water were added, and samples vortexed and shaken, centrifuged, and the supernatant aspirated and stored for subsequent analysis. Other sterile body fluids did not require special pre-treatment procedures.

##### DNA extraction

A 300-µL mixture of the pre-treatment products of each sterile body fluid sample was used with an internal reference. Then, 10 µL of protease K was added, and the sample was briefly centrifuged. Then, 300 µL of lysis solution was added, and the mixture was incubated at 70°C for 15 min and then rested at 4°C for 5 min. Next, 300 µL of anhydrous ethanol cooled to −20°C was added, and the mixture was inverted and placed for 5 min to permit instant separation. The supernatant was transferred to an adsorption column and centrifuged at 8,000 rpm for 30 s. The waste liquid was washed three times, centrifuged at 12,000 rpm for 2 min and dried for 3 min. Then, 45 µL of eluent was added, and the mixture was left to stand at room temperature for 5 min before being centrifuged at 12,000 rpm for 2 min. The eluted product was nucleic acid. The nucleic acid extraction and purification kit (RM0184, BGI, China) was used for this procedure. Then, the DNA concentration was measured using a Qubit fluorometer (Thermo Fisher, USA), and a DNA concentration >0.1 ng/µL was used for the next step.

##### Library construction and sequencing

A library was constructed by DNA fragmentation, end repair, linker connections, and PCR. A Qubit dsDNA HS assay kit (Thermo Fisher Scientific, Inc.) was used to quantify the concentration of the DNA library. The constructed library was pooled and cyclized, and DNA nanoballs (DNBs) were generated by rolling ring replication (RCA). The prepared DNBs were loaded onto a sequencing chip and sequenced using the MGISEQ-2000 platform. The sequencing strategy was a single-end 50 bp. At least 20 M of raw data were obtained for each sample.

##### Bioinformatics analysis

Low-quality data with a length of <35 bp were removed to obtain high-quality data. Via BWA alignment (http://bio-bwa.sourceforge.net/), human reference genome sequences were removed from the high-quality data. The remaining data after removal of low-complexity reads were classified by simultaneously aligning to the pathogens metagenomics database (PMDB), consisting of bacteria, fungi, viruses, and parasites. The classification reference databases were downloaded from National Center for Biotechnology Information (NCBI) (ftp://ftp.ncbi.nlm.nih.gov/genomes/). Based on the number of sequences, other laboratory-related examinations and comprehensive clinical information, such as imaging, and possible pathogens were identified.

### Culture testing

Bilateral double bottles of 8–10 mL of blood in each were collected from adults, and 1% blood of the total blood volume was collected from children for blood culture. Specialist doctors standardized the collection of the first tube of CSF, joint fluid, pleural fluid, ascites, tissue fluid, and pericardial effusion for cultivation. According to the bacteria culture procedures of the Microbiology Laboratory, routine separation media were used. Chocolate AGAR and blood AGAR plates were incubated in 5% CO2 at 37°C for 18–24 h. After bacteria grew on the agar medium, they were identified using matrix-assisted laser desorption ionization-time of flight mass spectrometry (VITEK MS, BioMerieux, France).

### Histopathology testing

The histopathology laboratory used standard methods for tissue samples.

### Statistical analysis

Demographic data are summarized using descriptive statistics and expressed as counts with percentages or medians with interquartile ranges. Infection-related inflammatory markers were analyzed using the Mann–Whitney *U* test, assuming that the observations were not normally distributed. Continuous variables were expressed as means with SDs and were compared using a *t*-test. Statistical tests were performed using GraphPad version 8.0.1 (GraphPad Software, San Diego, CA, USA) with a *P*-value ≤0.05 considered a significant difference.

## RESULTS

### Clinical characteristics of patients with negative mNGS results

**Fig 1:**
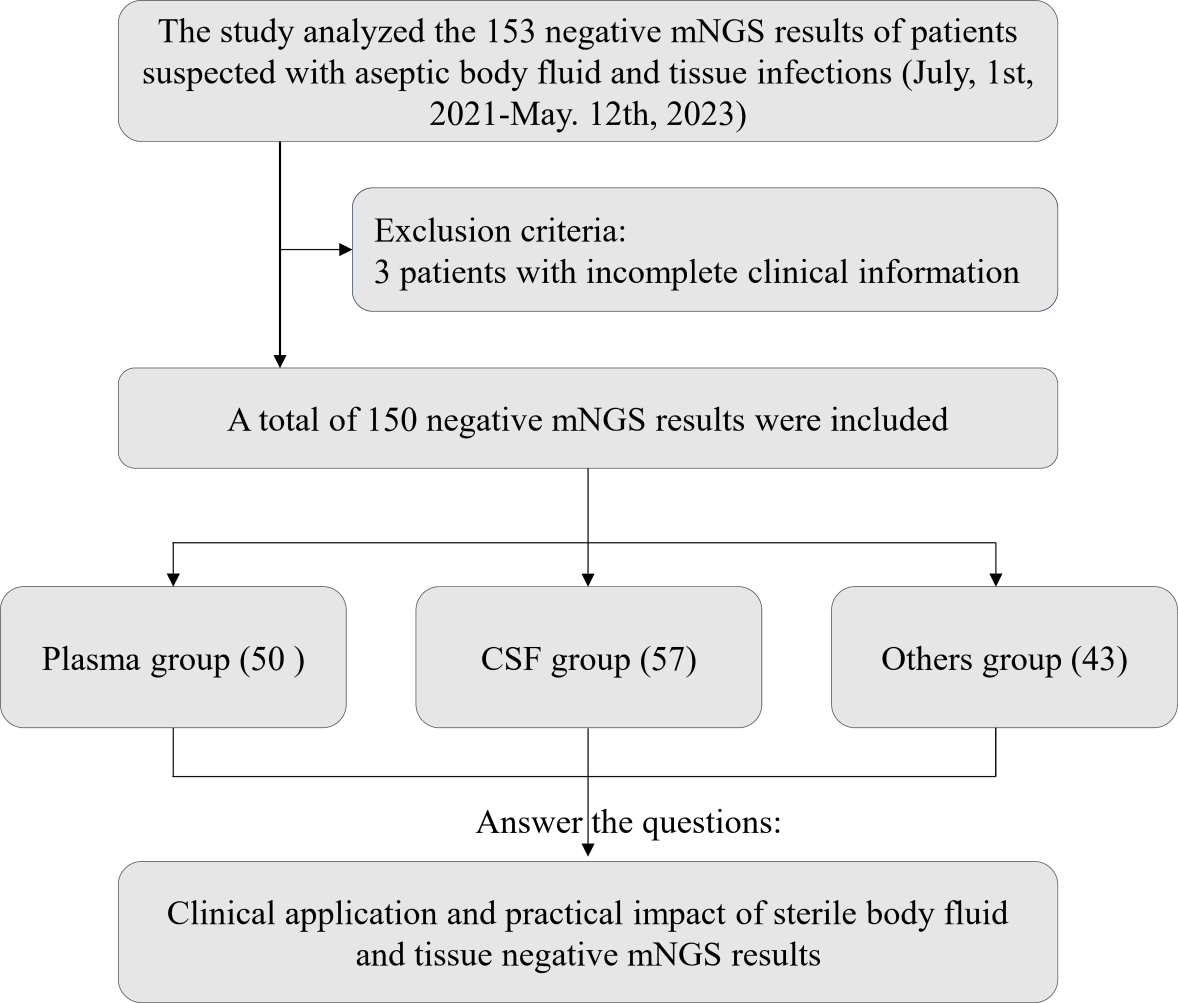
Patient enrollment workflow of 150 sterile body fluid and tissue samples. Abbreviations: mNGS, metagenomics next-generation sequencing; CSF, cerebrospinal fluid.

In the past 3 years, 150 negative mNGS reports were collected (Fig. 1) of sterile body fluids and tissues, of which 50 were assigned to a plasma group, 57 a CSF group, and 43 to others group (including 12 tissue fluids, nine joint fluids, nine tissues, six pleura effusion, five ascites, and two pericardial effusions). Among them, 18.0% of patients were in the intensive care unit (ICU), and a total of 56.0% of patients were male, with the median age being 57.5 years old. The admission diagnoses of the plasma group were trauma (18.0%), sepsis (24.0%), and fever (24.0%). Those of the CSF group were mainly intracranial infections (42.1%) and neurological symptoms (22.8%), whereas nine patients with joint fluid mNGS testing were mainly admitted for postoperative orthopedic infections (66.7%). A total of 104 (69.3%) patients received empirical antibacterial treatment before the mNGS trial (Table 1).

**TABLE 1:** Demographic and clinical characteristics of patients^*a*^

The study cohort	Plasma, *n* (%)	CSF, *n* (%)	Others, *n* (%)
No. of patients	50 (33.3)	57 (38.0)	43 (28.7)
Age, median years (range)	32.8 (1.0, 89.0)	33.0 (14.0, 89.0)	49.5 (21.0, 84.0)
Male	29 (58.0)	35 (61.4)	20 (46.5)
ICU	9 (18.0)	16 (28.1)	2 (4.7)
Admission diagnosis			
Trauma	9 (18.0)	1 (1.8)	1 (2.3)
Sepsis	12 (24.0)	2 (3.5)	2 (4.7)
Nervous system disease	/^*b*^	13 (22.8)	/
Cardiovascular disease	2 (4.0)	2 (3.5)	2 (4.7)
Cancer	1 (2.0)	2 (3.5)	2 (4.7)
Autoimmune disease	1 (2.0)	2 (3.5)	/
Unknown fever	12 (24.0)	2 (3.5)	3 (7.0)
Infection			
Pneumonia	5 (10.0)	1 (1.8)	1 (2.3)
Encephalitis	/	24 (42.1)	/
Urinary tract infection	1 (2.0)	/	/
Infection after orthopedic surgery	/	/	6 (14.0)
Spinal infection	/	/	1 (2.3)
Unclassified	7 (14.0)	8 (14.0)	25 (58.1)
Empirical treatment before mNGS testing	42 (84.0)	37 (64.9)	25 (58.1)

^*a*^ Abbreviations: CSF, cerebrospinal fluid; ICU, intensive care unit; mNGS, metagenomic next-generation sequencing.

^*b*^ “/”, This project has no patients.

### Clinical judgment of true negative, false negative, and uncertain negative results of mNGS

**Fig 2:**
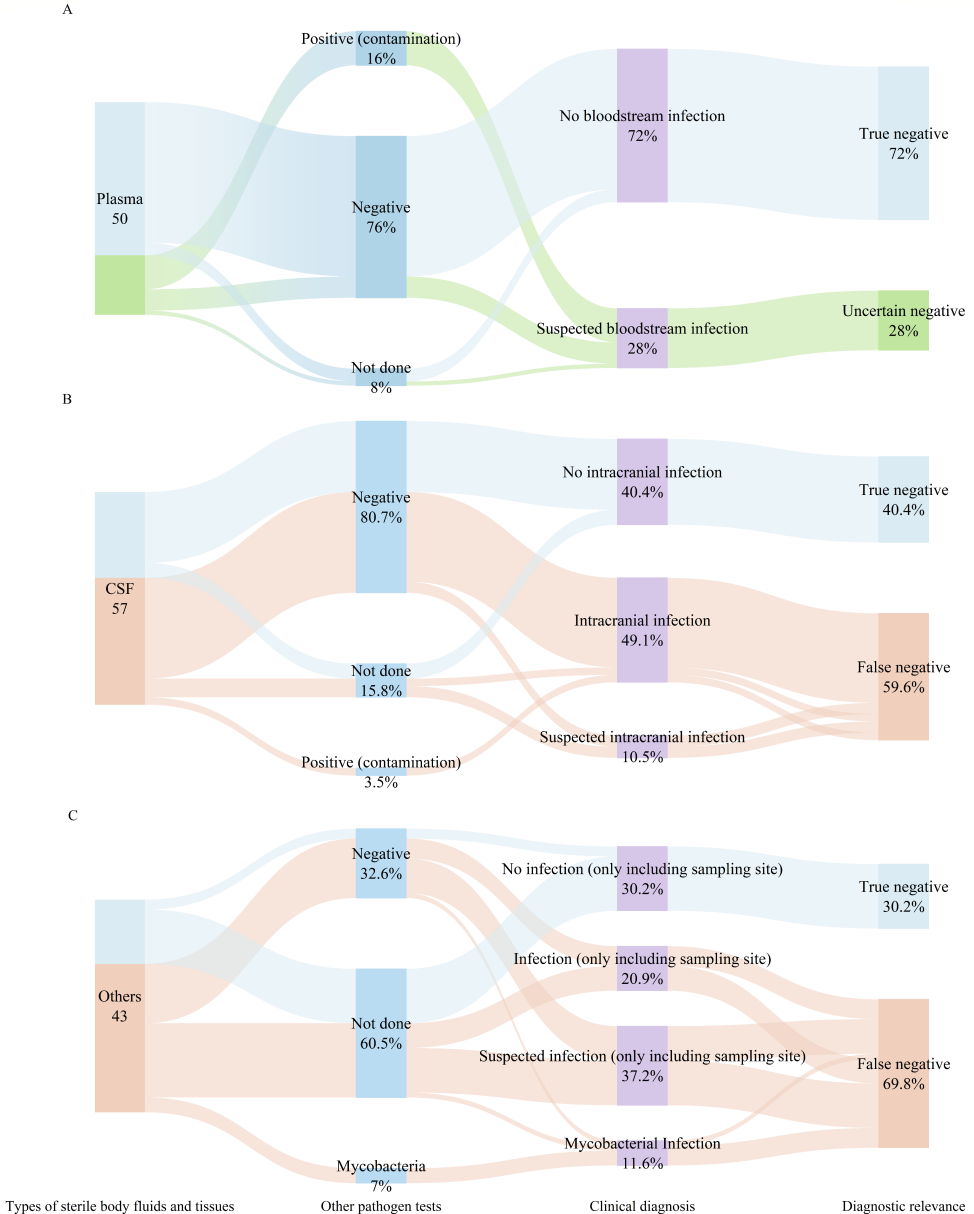
The clinical judgment of true negative, false negative, and uncertain negatives on (**A**) plasma, (**B**) CSF, and (**C**) other mNGS negative results. The blue connecting lines represent the clinical relevance validation process of mNGS true-negative patients with different sterile body fluids and tissues. The orange connecting lines represent the clinical relevance validation process of mNGS false-negative patients. The green connecting lines represent the clinical relevance validation process for mNGS uncertain-negative patients.

To clarify the negative mNGS results more clearly, we classified mNGS negative results based on other pathogen tests and clinical diagnosis of the patients (Fig. 2). Among all negative mNGS tests, 48% were true negative cases, 42.7% were false negative cases, and 9.3% were uncertain negative cases. The negative predictive values for negative mNGS results in plasma, CSF, and others groups were 72.0%, 40.4% and 30.2%, respectively.

In the plasma group, 46 (92%) patients also underwent blood culture, with 38 (76%) negative blood culture results and 8 (16%) positive results. According to the positive blood culture results, eight pathogens were identified including *Staphylococcus epidermidis* (three), *Staphylococcus haemolyticus* (two), *Corynebacterium striatum* (one), *Staphylococcus hominis* subsp. (one), and *Enterococcus faecalis* (one). However, after comprehensive judgment by the treatment team, the aforementioned eight cases of positive blood culture results were suspected to be contaminated and not considered pathogens (16). Due to the serious condition and high inflammatory index procalcitonin of the patients, the diagnosis was sepsis and severe pneumonia. Nevertheless, the treatment team still considered the possibility of a bloodstream infection. Therefore, we referred to these eight cases as uncertain-negative results.

For the CSF group, 48 (84.2%) patients underwent CSF culture, of which 46 (80.7%) were negative culture results and 2 (3.5%) were positive results. *Staphylococcus epidermidis* was detected in wo cases in the CSF culture group, but were considered contaminants. The treatment team diagnosed the patients with viral encephalitis, and the patients improved after antiviral treatments.

Among the others group, 9 (20.9%) also underwent culture with 100% being negative, and eight samples underwent histopathological tests. There were five suspected cases of tuberculosis infection, with three cases confirmed by histopathology and two cases supported by T-SPOT.TB detection. Finally, all the patients improved after empirical anti-tuberculosis treatment (Table 2).

**TABLE 2:** Clinical analysis of others' mNGS false-negative results in suspected *Mycobacterium* infection

	Age	Gender	Specimen type	Department	Clinical diagnosis	mNGS results	*Mycobacterium tuberculosis* culture	Chip method	Xpert	T-SPOT.TB detection	Histopathology	Treatment	Outcome
T1	63	Female	Tissue	Cardiothoracic surgery	Mycobacteria	Negative	/^*a*^	/	/	/	Positive	Anti-tuberculosis treatment	Improved discharge
T2	57	Male	Hydrothorax	Cardiothoracic surgery	Mycobacteria	Negative	/	Negative	/	/	Positive	Anti-tuberculosis treatment	Improved discharge
T3	70	Female	Tissue	Cardiothoracic surgery	Mycobacteria	Negative	Negative	/	Negative	Positive	Positive	Anti-tuberculosis treatment	Improved discharge
T4	69	Female	Tissue	Orthopedics	Suspected mycobacteria	Negative	/	/	/	Positive	/	Empirical anti-tuberculosis treatment	Improved discharge
T5	55	Female	Pericardial effusion	Cardiology department	Mycobacteria	Negative	Negative	Negative	Negative	Positive	/	Anti-tuberculosis treatment	Improved discharge

^*a*^ “/”, This project has no patients.

### The impact of negative mNGS results on patient diagnosis, treatment, and outcomes

According to the definition of clinical impact in the present study, the plasma, CSF, and other negative mNGS results showed a positive clinical impact on 34 patients (68.0%), 23 patients (40.4%), and 11 patients (25.6%), respectively (Table 3). In contrast, negative results of 16 (32.0%) plasma samples, 34 (59.6%) CSF samples, and 32 (74.4%) others samples did not affect the clinical management of patients.

**TABLE 3:** Clinical impact of negative mNGS results in sterile body fluids and tissues (*n* = 150)

	Clinical impact classification		Case number (%)	Change treatment (%)	Infection after excluding mNGS sampling site infection	Improved outcome (%)
Plasma (50)	Positive impact	Excluded bloodstream infection	34 (68.0%)	3/34 (8.8%)	28/34 (82.4%)	28/34 (82.4%)
	No impact	A negative result with no clinical significance	12 (24.0%)	/^*a*^	/	9/12 (75.0%)
		Patient forgoes further treatment (discharge or death)	4 (8.0%)	/	/	0/4 (0.0%)
CSF (57)	Positive impact	Excluded intracranial infection	23 (40.4%)	4/23 (17.4%)	10/23 (43.5%)	17/23 (73.9%)
	No impact	A negative result with no clinical significance	28 (49.1%)	/	/	25/28 (89.3%)
		Patient forgoes further treatment (discharge or death)	6 (10.5%)	/	/	3/6 (50.0%)
Others (43)	Positive impact	Excluded infection (only includes sampling site)	11 (25.6%)	/	6/11 (54.5%)	10/11 (90.9%)
	No impact	A negative result with no clinical significance	28 (65.1%)	/	/	25/28 (89.3%)
		Patient forgoes further treatment (discharge or death)	4 (9.3%)	/	/	2/4 (50.0%)

^*a*^ “/”, This project has no patients.

The impact of mNGS negative results on patients was not only reflected in the diagnosis and exclusion of infection but also in the clarification of patient treatment and, most importantly, in the improvement of patient outcomes. The treatment status and outcome distribution of patients before and after mNGS testing are listed in Table 4. There were three patients (6.0%) in the plasma group who had changed treatment based on mNGS negative results, with 66.7% of patients improving; 43 patients (86.0%) did not change the treatment before and after mNGS testing, and 81.4% improved; a total of four patients (8.0%) were discharged before reporting mNGS. There were four patients (7.0%) in the CSF group who had a change in their treatment based on mNGS negative results, with only 25.0% of the patients improving; 47 patients (82.5%) did not change their treatment before and after mNGS testing, with 87.2% of the patients improving. There were 39 patients (90.7%) in the others group who did not change their treatment before and after mNGS testing, and 89.7% of the patients improved. Four patients (9.3%) were discharged before reporting mNGS.

**TABLE 4:** The impact of mNGS negative results on patient treatment and outcomes (*n* = 150)

	The treatment type (n)			Improved outcome (%)
Plasma (50)		Pre-mNGS	Post-mNGS	
	Not change the treatment (43)	Antibiotic (38)	Same antibiotics	30 (78.9%)
		Symptomatic treatment without antibiotics (5)	Symptomatic treatment without antibiotics	5 (100.0%)
	Change the treatment (3)	Cefoperazone, doxycycline	Cefoperazone	1 (100.0%)
		Imipenem, linezolid	Moxifloxacin	1 (100.0%)
		Cefoperazone	Hormones	0 (0.0%)
	Uncertain treatment (4)	Patient discharge	/^*a*^	0 (0.0%)
CSF (57)	Not change the treatment (47)	Antibiotic (39)	Same antibiotics	33 (84.6%)
		Symptomatic treatment without antibiotics (8)	Symptomatic treatment without antibiotics	8 (100.0%)
	Change the treatment (4)	Vancomycin, meropenem	Cefoperazone	0 (0.0%)
		Ganciclovir	Symptomatic treatment without antibiotics	1 (100.0%)
		Ceftriaxone, acyclovir	Acyclovir	0 (0.0%)
		Ceftriaxone, acyclovir	Acyclovir	0 (0.0%)
	Uncertain treatment (6)	Patient discharge	/	3 (50.0%)
Others (43)	Not change the treatment (39)	Antibiotic (32)	Same antibiotics	29 (90.6%)
		Symptomatic treatment without antibiotics (7)	Symptomatic treatment without antibiotics	6 (85.7%)
	Uncertain treatment (4)	Patient discharge	/	2 (50.0%)

^*a*^ “/”, This project has no patients.

### The related factors of negative results in mNGS tests

**Fig 3:**
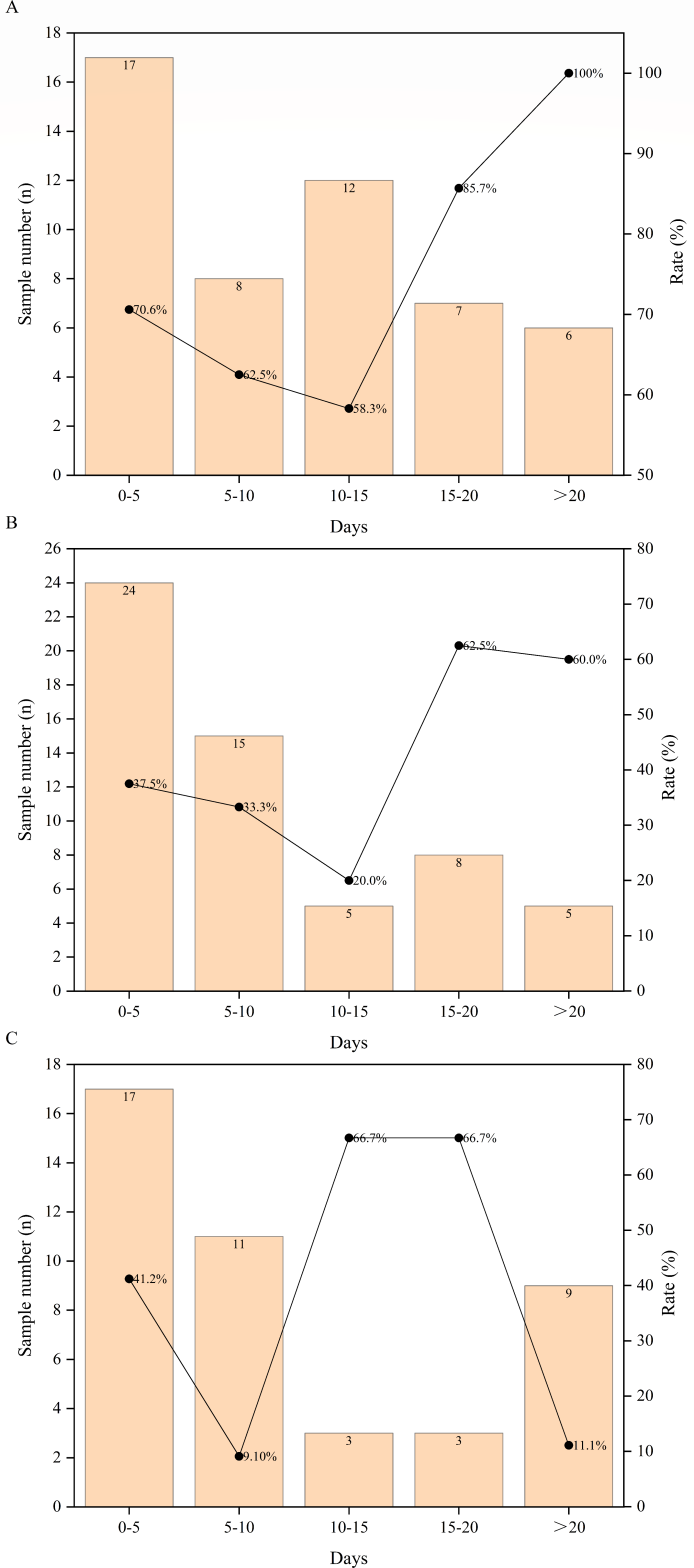
Sampling timing analysis of mNGS tests. Analysis of the sampling timing of mNGS on (**A**) plasma, (**B**) CSF, and (**C**) other mNGS negative results. The left vertical axis represents the sample number, represented by a bar chart; the right vertical axis represents the true-negative rate distribution of patients in different time groups from the appearance of suspected infection symptoms to mNGS sampling, represented by a line graph.

We analyzed the optimal timing for mNGS sampling after patients showed suspected symptoms of infection (Fig. 3). Within half a month after the appearance of suspected infection symptoms, the true-negative rate also decreased over time. In other groups, due to the small sample size, and in the plasma and CSF groups, the true-negative rate significantly increased after more than half a month of suspected infection symptoms, possibly due to the small sample size and the fact that the patients were not infected.

**Fig 4:**
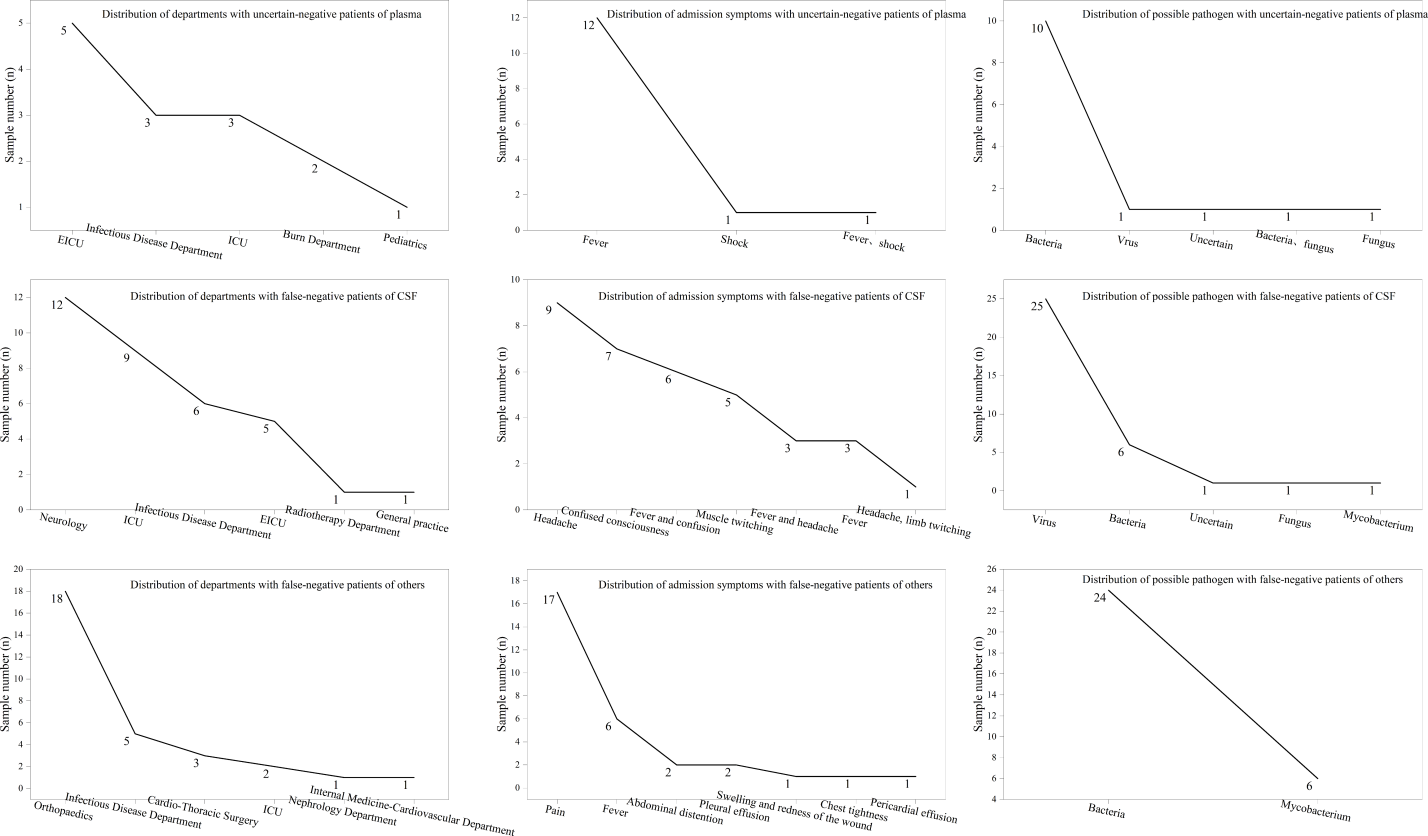
Clinical information analysis of mNGS uncertain-negative and false-negative results. Analysis of the department distribution, admission symptom distribution, and possible pathogen analysis of plasma, CSF, and other mNGS uncertain-negative and false-negative patients. Abbreviations: ICU, intensive care unit; EICU, emergency intensive care unit.

At the same time, we analyzed the clinically relevant information of mNGS false-negative and uncertain-negative patients (Fig. 4). Plasma mNGS uncertain-negative patients mainly came from emergency intensive care unit (EICU) (35.7%), infectious disease department (21.4%) or the intensive care unit (ICU) (21.4%); uncertain-negative patients were admitted mainly due to fever (92.9%). The doctor’s clinical judgment showed that 71.4% of uncertain-negative patients had bacterial infections. CSF mNGS false-negative patients mainly came from the ICU (26.5%) or neurology (35.3%), and false-negative patients were admitted mainly due to headache (38.2%) and fever (35.2%). The doctor’s clinical judgment showed that 73.3% of false-negative patients had viral meningitis. Others group mNGS false-negative patients mainly came from orthopedics (60%) or infectious disease departments (16.7%), and false-negative patients were admitted mainly due to pain (53.3%) and fever (20.1%). Doctors clinically determined that 80% of the other mNGS false-negative patients may have had bacterial infections.

## DISCUSSION

In the present study, the correlation between negative mNGS results in the plasma, CSF, and other groups was analyzed as well as the patients' condition and the impact on the doctor’s diagnosis and treatment. We also conducted an in-depth analysis of the effect of the time from symptom onset to mNGS sampling on mNGS negative results in suspected aseptic fluid infection patients. Finally, we analyzed the possible reasons for false-negative patients in terms of admission symptoms, department analysis, and the doctor’s perception of the pathogen that the patient may be infected with.

In our study, the diagnostic accuracy of excluding infection in the plasma, CSF, and others groups was 72.0%, 40.4%, and 30.2%, respectively. However, only three cases in the plasma group and four cases in the CSF group required a change in their treatment regimen after a clear diagnosis. The Hogan team established a standardized evaluation of the clinical impact of plasma mNGS, while the Feng team analyzed the impact of mNGS on patient diagnosis and treatment for hematological diseases (17, 18). More and more studies have shown that mNGS needs to complement other pathogen detection techniques, combined with the patient’s condition and the doctor’s judgment, for its role as an auxiliary diagnostic tool to be fully recognized. This was also one of our research objectives, namely, to expand the clinical application of mNGS by analyzing the negative results of mNGS.

There are several challenges in interpreting negative mNGS results in clinical practice. The most vigorous clinical interpretation is that accurate negative results unfold good negative predictive value for excluding infections. However, for certain pathogenic microorganisms, such as *Mycobacterium tuberculosis*, which have a low content in the original samples or are difficult to extract nucleic acids from, it should be considered that mNGS tests show low sensitivity and poor negative predictive ability, and may not necessarily be superior to conventional detection schemes such as PCR (5, 19–21). Wilson et al. reported that in 18 patients with meningitis or encephalitis who tested negative for CSF using mNGS, the diagnosis was confirmed through serological experiments (13).

mNGS false-negative results indicated that the patients still had pathogenic infections, but mNGS failed to detect them. This insufficient detection was likely due to the pathogen’s inherent characteristics, such as the thick outer wall of some pathogenic microorganisms or difficulty in breaking the wall during nucleic acid extraction, resulting in incomplete release of nucleic acids, as seen in *Aspergillus*, *Mucor*, *Cryptococcus*, *Nocardia,* and *Mycobacterium* (19, 20). Also, the database for interpreting mNGS results may have been incomplete and the load of pathogenic microorganisms in a sample lower than the minimum detection limit of mNGS (22). In short, a single negative mNGS result cannot be used to make clinical decisions and must be comprehensively and dynamically analyzed.

In 2019, the Miller research group published a laboratory validation article for pathogens assessed using mNGS tests, with PCR to validate CSF negative mNGS samples (9). Another study validated mNGS false negatives through cultivation (23). The present study has analyzed the reasons for mNGS false negatives in clinical applications. When mNGS results were negative and pathogens were detected in plasma and CSF cultures, they may not necessarily be pathogenic, but may be contaminations during sampling or operations (4). In clinical practice, it is necessary to comprehensively judge whether negative mNGS results are false negatives based on the patient’s symptoms.

The present study had a number of limitations. It only analyzed the reasons for mNGS false negatives from a clinical application perspective. We did not integrate the validation of other laboratory testing techniques to analyze deeply the reasons for false negatives more comprehensively and improve the accuracy of mNGS diagnosis. In addition, the research did not delve deeper into practical problem-solving methods, such as specific improvement strategies for departments with high false-negative rates and clear procedures for improving true-negative results.

In conclusion, the study analyzed the actual impact of negative mNGS results with sterile body fluids and tissues in patients considering clinical applications. The true-negative rate of mNGS tests in the plasma group was the highest. We statistically analyzed that from the appearance of suspected infection symptoms in patients to mNGS sampling, the true-negative rate of mNGS decreased significantly with the prolongation of time within half a month. We recommend early mNGS testing for suspected infected patients in clinical practice. The false-negative rates of the CSF and others groups were high. The study analyzed the reasons from the following three aspects: patient department distribution, admission symptoms, and clinical judgment of patients' conditions. We believe that mNGS can play a greater role in the clinical application of infection diagnosis.

## Data Availability

The datasets CNSA for this study can be found at CNP0006560 [https://db.cngb.org/mycngbdb/submissions/project].
